# Blastocyst-Bearing Sows Display a Dominant Anti-Inflammatory Cytokine Profile Compared to Cyclic Sows at Day 6 of the Cycle

**DOI:** 10.3390/ani10112028

**Published:** 2020-11-04

**Authors:** Inmaculada Parrilla, Cristina A. Martinez, Josep M. Cambra, Xiomara Lucas, Graça Ferreira-Dias, Heriberto Rodriguez-Martinez, Cristina Cuello, Maria A. Gil, Emilio A. Martinez

**Affiliations:** 1 Faculty of Veterinary Medicine, International Excellence Campus for Higher Education and Research “Campus Mare Nostrum”, University of Murcia, 30071 Murcia, Spain; josepmiquel.cambra@um.es (J.M.C.); xiolucas@um.es (X.L.); ccuello@um.es (C.C.); mariagil@um.es (M.A.G.); emilio@um.es (E.A.M.); 2 Campus de Ciencias de la Salud, Institute for Biomedical Research of Murcia (IMIB-Arrixaca), 30071 Murcia, Spain; 3 Department of Biomedical & Clinical Sciences (BKV), BKH/Obstetrics & Gynaecology, Faculty of Medicine and Health Sciences, Linköping University, 58183 Linköping, Sweden; heriberto.rodriguez-martinez@liu.se; 4 CIISA—Centre for Interdisciplinary Research in Animal Health, Faculty of Veterinary Medicine, University of Lisbon, 1649-004 Lisbon, Portugal; gmlfdias@fmv.ulisboa.pt

**Keywords:** endometrium, explants, cytokines, embryo transfer, maternal immune response, pig

## Abstract

**Simple Summary:**

A proper uterine environment is basic for obtaining optimal embryo transfer outputs in domestic species, including the pig. However, scarce information is available about the uterine immune response of recipient (uninseminated) sows when receiving embryos during embryo transfer. Endometrial cytokine profile is among the main factors regulating uterine receptivity to embryos. In this study, using Luminex MAP^®^ technology, we found important differences in the endometrial production in most of the 16 cytokines analyzed between recipient sows and embryo-bearing (inseminated) sows six days after estrus, with a predominant cytokine anti-inflammatory environment in the embryo-bearing endometria. These observations suggest that insemination components and/or early embryos induce an endometrium immune-tolerant cytokine profile at Day 6 of the cycle. The findings could contribute importantly to design strategies to maximize the reproductive performance of recipients after embryo transfer in swine.

**Abstract:**

In the context of porcine embryo transfer (ET) technology, understanding the tightly regulated local uterine immune environment is crucial to achieve an adequate interaction between the transferred embryos and the receiving endometrium. However, information is limited on the uterine immune status of cyclic-recipient sows when receiving embryos during ET. The present study postulated that the anti- and proinflammatory cytokine profile 6 days after the onset of estrus differs between endometria from uninseminated cyclic sows and blastocyst-bearing sows. On Day 6 of the cycle, endometrial explants were collected from sows inseminated or not inseminated during the postweaning estrus and cultured for 22 h. The culture medium was then analyzed for the contents of a total of 16 cytokines using Luminex MAP^®^ technology. The results showed important differences in the endometrial production of most cytokines between the sow categories, with a predominant anti-inflammatory environment displayed by the blastocyst-bearing endometria. These findings suggest that sperm, seminal plasma (SP) and/or early embryos modify the uterine environment by inducing an immune-tolerant cytokine profile already visible at Day 6. Whether the SP or some of its active components may help to develop strategies to maximize the reproductive performance of recipients after ET needs further investigation.

## 1. Introduction

Embryo transfer (ET) technology is a powerful tool to accelerate livestock genetic improvement by transferring embryos with highly valuable genetics while avoiding the risk of transmitting diseases, reducing transport costs of livestock, and preventing animal welfare aspects related to transportation [1]. Beyond these important productive benefits, ET is the decisive last step for other biotechnologies, including cloning, transgenesis, zygote gene editing and blastocyst complementation [2]. Despite these important advantages, the use of ET in pigs has not reached the levels attained in other domestic animals, such as cattle. Although the main drawback for a broad application of ET in pigs comes from the necessity of using surgical methods to retrieve embryos, a lower litter size is also a determinant for its limited use [2,3]. Approximately 55% of transferred embryos die before, during, or shortly after implantation in the reproductive tract of recipients after ET, values almost 25 percentage points higher than those obtained by artificial insemination (AI) [4].

It is well known that under physiological conditions in pigs, AI components, including seminal plasma (SP) and spermatozoa, provoke a rapid uterine inflammatory response that induces changes at the cellular and molecular levels [5,6,7,8]. This immediate endometrial inflammatory reaction post-insemination is transient and quickly gives rise to an anti-inflammatory immunotolerant environment that favors embryo survival and proper progression of pregnancy [8,9,10,11,12]. Additionally, the female reproductive tract is able to detect embryo presence prior to implantation and to accordingly modify its immune response [13,14] towards successful implantation [15]. Cytokines are major immune regulators linked to optimal endometrium receptivity and embryo growth [16,17,18]. The SP, spermatozoa and developing zygotes all contribute to modulating cytokine signaling in the female reproductive tract [4,19,20,21,22]. Therefore, their presence should be crucial for optimal pregnancy outcomes. This is an important aspect in the context of porcine ET technology, especially regarding the recipient females, which under standard conditions, are not subjected to some of these stimuli before ET. It could be expected that in the absence of these preparative factors, the uterine environment of recipients is not the most adequate for embryo reception, development, and survival after ET. Supporting this hypothesis, a faulty communication between embryos and endometrium is a determinant factor for failures in embryo progression and subsequent implantation [23]. Moreover, in pigs, an asynchrony of more than 24 h in the endometrial status between donors (embryos) and recipients notably decreases ET-pregnancy rates [24], indicating that fine tuning of the temporal regulation of the maternal immune system is required to achieve adequate conceptus growth [25].

Despite the importance that immune mechanisms seem to have at these very early stages, information about the endometrial immune status of ET and cyclic sows around days 5–6 is limited. A better understanding of the molecular mechanisms modulating the uterine immune environment during these days could be essential for the improvement of ET technology, since those are the days generally used for transferring the embryos into recipients [12].

The general aim of this study was to deepen our knowledge of maternal immune mechanisms that regulate the preimplantation period. In particular, the study focused on the endometrial profiles of 16 anti- and proinflammatory cytokines of blastocyst-bearing (BB) and uninseminated cyclic (U-IC) sows at Day 6 of the cycle.

## 2. Materials and Methods

### 2.1. Animals

The experiment was carried out following the directive 2010/63/EU and was evaluated and approved by the Murcia University Ethical Committee for Animal Experimentation (Spain) (ref. 22072015) and the Autonomous Government of Murcia (Spain) (ref. 01062016/125089).

Weaned Landrace × Large-White multiparous sows (3 to 4 parity) were used for the experiment. The sows were individually located in the gestation room of a production farm (Agropor SA, Murcia, Spain) with controlled temperature and ventilation. The animals were fed standard diets depending on their nutritional needs and had free access to water. Insemination doses from boars of proven fertility were bought from a commercial AI station (AIM Iberica, Murcia, Spain).

### 2.2. Synchronization, Detection of Estrus, and Insemination

The weaning of lactating sows was used for estrus synchronization among the sows. Only sows in estrus 4 or 5 days after weaning were used in the experiment. The detection of estrus was carried out by experienced operators in the presence of a boar twice a day, starting 24 h after weaning. Sows exhibiting a clear standing reflex were considered in estrus, and Day 0 of the cycle was defined as the first day of estrus. Inseminations were performed using the post-cervical AI procedure [26] at 6 h and 24 h after the onset of estrus with doses containing 1.5 × 10^9^ spermatozoa in 40 mL of Beltsville Thawing Solution (BTS) extender [27]. All sows were inseminated with pooled sperm doses from same three boars kept at 18 °C for less than 24 h.

### 2.3. Embryo Collection

Embryos were retrieved on Day 6 from inseminated sows as previously reported [3]. Sedation and general anesthesia were achieved by azaperone (i.m.; 2 mg/kg of body weight; Stresnil^®^, Sanochemia Pharmazeutika AG. Neufeld/Leitha, Austria) and sodium thiopental (i.v.; 7 mg/kg of body weight; B. Braun VetCare SA, Barcelona, Spain). General anesthesia was continued with isoflurane (3.5–5% in oxygen; IsoFlo^®^, Madrid, Spain). The embryos were retrieved from the tip of each uterine horn (30 cm from the utero-tubal junction) with 30 mL of washing medium [28]. Immediately after washing, the morphological quality and developmental stage of embryos were evaluated under stereomicroscopy. Embryos with appropriate morphology and developmental stage according to the criteria of the International Embryo Transfer Society [29] were considered viable.

### 2.4. Tissue Collection

Sows were hysterectomized on Day 6 of the cycle. The uterine tracts were transported on ice to the laboratory within 2 h of collection. Then, each uterine horn was opened longitudinally along the anti-mesometrial side, and endometrial samples from the distal (ad-cervical), middle portion and proximal (ad-ovarian) regions of each uterine horn were obtained from each sow.

### 2.5. Culture of Endometrial Explants

Working under sterile conditions, small sections (2–3 mm^3^) of endometrium were washed in PBS supplemented with gentamicin (20 μg/mL), and a total of three sections of tissue (40 mg/mL) were placed in 3 mL of NCSU-23 medium [30]. The explants were cultured with agitation (150 rpm) at 38 °C in 5% CO_2_ in air for 1 h and then in fresh culture medium under the same conditions for another 22 h. Explants from each uterine region (three regions, six explants per sow) were cultured in triplicate, and the media collected from each uterine region were pooled, centrifuged (13,000× *g*; 5 min) to eliminate debris and kept at −80 °C until cytokine analysis. The duration of culture was 22 h, which is within the innate immune response. Lactate dehydrogenase (LDH) activity in spent media was determined in an automated analyzer (AU 600, Olympus, Minneapolis, MN, USA) to assess the cell integrity of the explants at the beginning and end of the in vitro culture time. LDH activity at 0 h and 22 h of culture was 1.5 ± 0.6 mU/mg and 6.3 ± 1.7 mU/mg, respectively.

### 2.6. Cytokine Analysis

The concentration of a total of 16 cytokines was measured in supernatants of explant culture media by using the Luminex^®^ xMAP^®^ technology in combination with two Merck Millipore kits, namely the MILLIPLEX MAP Porcine Cytokine and Chemokine Magnetic Bead Panel-Immunology Multiplex Assay (PCYTMG-23K-13PX; Merck Millipore, Burlington, MA, USA), which allows the analysis of 13 analytes [granulocyte macrophage colony-stimulating factor (GM-CSF), interferon gamma (IFN-γ), interleukin (IL)-1α, IL-1ra, IL-1β, IL-2, IL-4, IL-6, IL-8, IL-10, IL-12, IL-18, and tumor necrosis factor α (TNFα)], and the MILLIPLEX MAP TGFβ Magnetic Bead 3 Plex Kit-Immunology Multiplex Assay (TGFBMAG-64K-03; Merck Millipore, Burlington, MA, USA) for quantification of transforming growth factor β (TGFβ) 1, TGFβ2 and TGFβ3.

All measurements were carried out following the guidelines provided by the manufacturer. Supernatant samples for TGFβ analyses were acidified (pH < 3) and diluted 1:2 before the analysis. The culture medium was used as the matrix solution in standard curve, control, and blank wells. Samples were run in duplicate, and kits and reagents belonging to the same lots were used throughout the entire study. Once sonicated, bead solution was added to each well and incubated in the dark (4 °C; 18 h) under continuous shaking. Then, the content of the wells was removed, and two or three washing steps (depending on the kit used) were performed using the washing solution included in the kits. Detection antibodies were then added to each well, and the plates were kept in darkness at room temperature for 60 min or 120 min (depending on the kit). Thereafter, streptavidin-phycoerythrin was added to each well, and the plates were incubated again (room temperature, 30 min). After this incubation period, the plates were washed and run in a MAGPIX R (Luminexcorp, Austin, TX, USA). Data were acquired using xPONENT software version 4.2 (Luminexcorp, Austin, TX, USA), and the data analysis was performed with MILLIPLEX R Analyst Version 5.1 (Merck Millipore, Darmstadt, Germany). Mean fluorescence intensities were analyzed using a 5-parameter logistic curve-fitting to determine the cytokine concentration (pg/mL) in supernatants.

### 2.7. Experimental Design

To evaluate endometrial cytokine production, 8 multiparous sows were assigned to one of two groups. (1) The BB group (N = 4): sows were inseminated during postweaning estrus. These sows were subjected to laparotomies to evaluate the presence of embryos in the uterus at Day 6 of the cycle (Day 0 = onset of estrus). (2) The U-IC group: sows were not inseminated during the postweaning estrus (N = 4). Endometrial explants were collected from all sows on Day 6 and cultured in vitro for 22 h for cytokine analysis of the culture medium. Explants were obtained from three different locations of each uterine horn (six samples per sow) and processed as described above.

### 2.8. Statistical Analysis

Statistical analysis was performed with SPSS Statistics version 19 (IBM SPSS Statistics, Chicago, IL, USA). The cytokine levels were analyzed using the Shapiro-Wilk test for normality followed by the nonparametric Kruskal-Wallis test (data analysis of the uterine areas) or Mann-Whitney U test (rest of the data analyses). Outliers were detected via boxplots and eliminated from the statistical analysis. The ratios between pro and anti-inflammatory cytokine in each group of sows (blastocyst-bearing sows and cyclic sows) were calculated by dividing the levels of anti-inflammatory cytokines by the proinflammatory cytokines of each sample. Differences between the two groups were analyzed by Student’s *t*-test or the Mann-Whitney U test for ratios normally and not normally distributed, respectively. A *p*-value <0.05 was considered to be statistically significant. The data are shown as the mean ± standard deviation (SD), median, and percentiles.

## 3. Results

All inseminated sows had more than 8 viable early embryos in each uterine horn. From a total of 76 structures recovered, 98.7% were embryos and 1.3% oocytes. All embryos collected were considered viable, and 90.7% and 9.3% of them were at the full and expanded blastocyst stages, respectively.

In this study, we determined the levels of cytokines produced by endometrial explants of BB and U-IC sows. The data obtained from the different uterine areas (distal, middle and proximal) and uterine horns (right and left) for each animal within each group were analyzed together because there were no significant differences between uterine regions.

**Figure 1:**
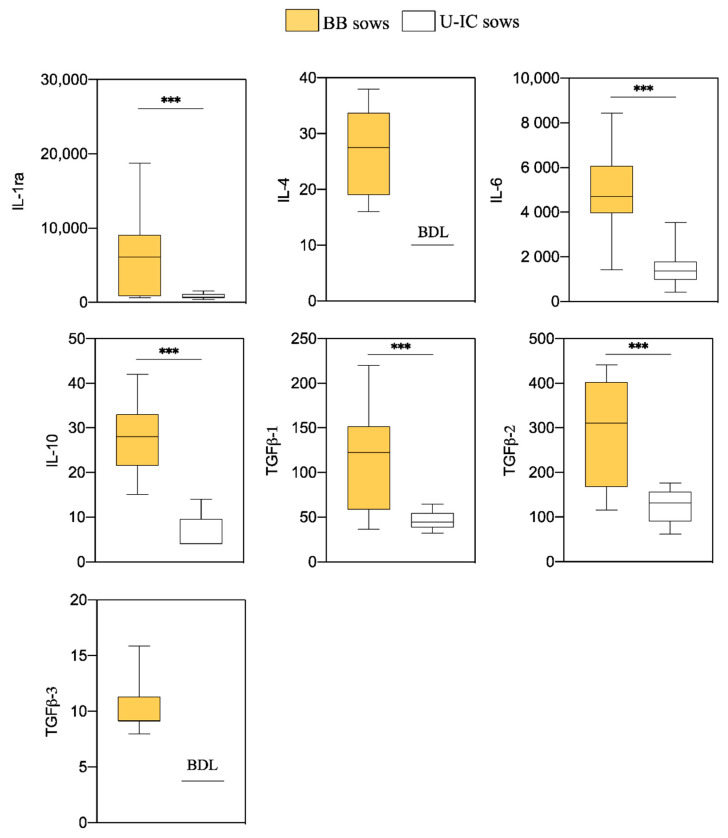
Differences in endometrial anti-inflammatory cytokine levels six days after the onset of estrus between blastocyst-bearing (BB) and uninseminated cyclic (U-IC) sows. Data are presented as box plots showing the median and interquartile range (Q1–Q3). BDL: values below the detection limits for the assay. Asterisks indicate significant differences between groups (*** *p* < 0.001).

**Figure 2:**
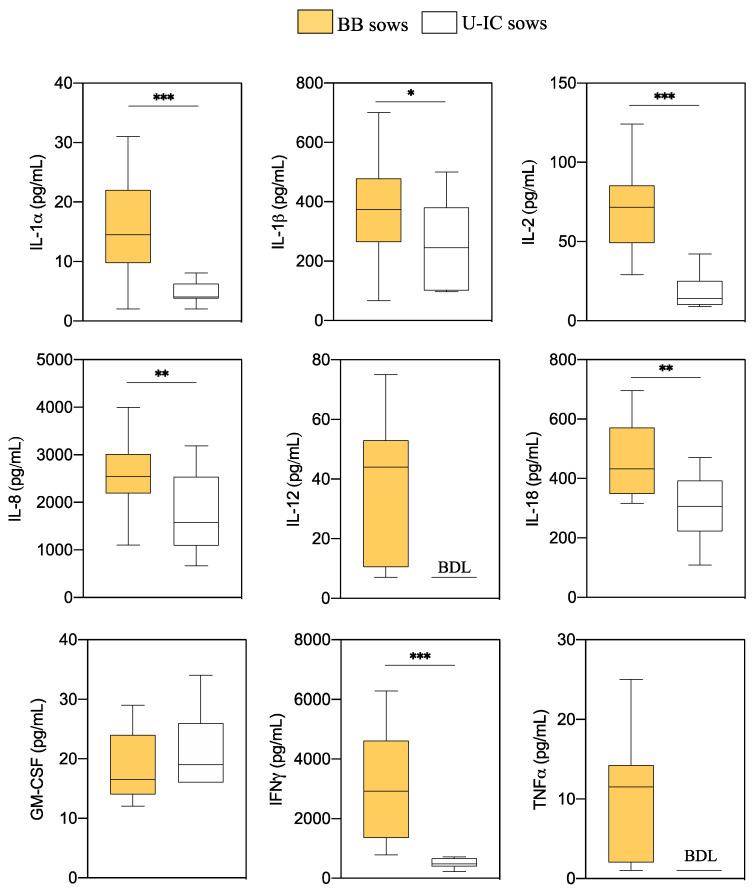
Differences in endometrial proinflammatory cytokine levels six days after the onset of estrus between blastocyst-bearing (BB) and uninseminated cyclic (U-IC) sows. Data are presented as box plots showing the median and interquartile range (Q1–Q3). BDL: values below the detection limits for the assay. Asterisks indicate significant differences between groups (* *p* < 0.05; ** *p* < 0.01; *** *p* < 0.001).

The anti- and proinflammatory cytokine concentrations detected in endometrial explants of BB and U-IC sows are presented in Figure 1 and Figure 2, respectively. In general, most of the anti- and proinflammatory cytokines were produced at higher levels in explants from BB sows compared to those of U-IC sows.

All the anti-inflammatory cytokines measured (IL-1ra, IL-4, IL-6, IL-10, TGFβ1, TGFβ2 and TGFβ3) showed measurable levels in BB sows, while in U-IC sows, the concentrations of IL-4 and TGFβ3 were below the detection limit of the assay, and they were not further analyzed. When the levels of IL-1ra, IL-6, IL-10, TGFβ1 and TGFβ2 were compared between both types of sows, higher (*p* < 0.001) values were obtained in explants from BB sows.

Measurable values of all proinflammatory cytokines analyzed (IL-1α, IL-1β, IL-2, IL-8, IL-12, IL-18, GM-CSF, IFNγ and TNFα) were obtained in endometrial explants of BB sows, while the levels of IL-12 and TNFα in U-IC sows were below the detection limits and were not further analyzed. All the proinflammatory cytokines evaluated, except GM-CSF, showed higher concentrations in BB than in U-IC sows (*p* < 0.05).

The ratios of anti- to proinflammatory cytokines showing significant differences (*p* < 0.05) between BB and U-IC sows are displayed in Table 1. A total of 25 ratios were significantly different between both groups evaluated. Fifteen of them (60%) were biased toward anti-inflammatory status in BB sows, whereas this percentage only reached 48% in U-IC sows. A total of 83.3% of the ratios that shifted towards anti-inflammatory status in both types of sows were significantly higher (*p* < 0.05) in BB than in U-IC sows.

## 4. Discussion

The present study shows important differences in endometrial cytokine production at Day 6 of the cycle between BB and U-IC sows, with a shift towards an anti-inflammatory response in BB endometria.

Before discussing the differences in uterine cytokine profile between BB and U-IC sows, there is an aspect that should be mentioned. Laparotomy procedure is a major surgery that induces a surgical stress modifying the individual’s immune response by different mechanism including alteration of cytokine secretion [31]. Therefore, laparotomy could affect cytokine secretion at uterine level under our experimental conditions. However, since both groups of sows (BB sows and U-IC sows) were subjected to the same surgical procedure and therefore to the same surgical stress the differences in uterine cytokine expression between both groups were more probably due to their different physiological status than to an inflammatory response to laparotomy procedure.

The fact that most of the cytokine concentrations and the ratios of anti- to proinflammatory cytokines were higher in BB than in U-IC sows suggests that these molecules are essential components of the uterine preparation for a potential pregnancy. The uteri of BB sows were exposed to several stimuli, including the AI components (SP and sperm) and the presence of embryos, which can change the endometrial inflammatory response [8,13,14,32]. There is some evidence supporting this suggestion. For instance, the effects of SP infused during estrus persist during the preimplantation period modifying the transcriptome at endometrial and embryonic levels by up-regulation of genes and pathways related to maternal immune tolerance and embryonic development, implantation and pregnancy development [12,33]. In the same way, it has been reported that the presence of blastocysts in the uterine horn alters many endometrial transcripts related to the maternal immune system, which could permit the non-receptive uterus to accept the embryos and their further development [14]. Our results suggest that in U-IC sows, the absence of these stimuli could have decreased the uterine immune response, resulting in the diminution of cytokine production by the uterus.

IL-1RA was one of the cytokines with the highest differences between BB and U-IC sows. This cytokine, whose abundance in BB sows increased almost 9-fold compared to U-IC sows, acts as a natural competitor of IL-1β inhibiting its proinflammatory response [34,35]. Pig blastocysts at Day 6 express IL-1 β2, the embryonic form of IL-1β [15], which has been related to the creation of a proinflammatory microenvironment in the endometrium at the peri-implantation period [15,23]. Thus, the high levels of IL-1RA in pregnant samples might be a response to the inflammatory reaction caused by blastocyst IL-1β2 secretion [35], which would favor an anti-inflammatory state to avoid the rejection of semi-allogenic embryos.

IL-6, a pleiotropic cytokine that can exert anti- and proinflammatory functions in a context-dependent manner [36], is considered one of the most relevant cytokines for successful pregnancy establishment in mammals, including pigs [15,37]. Interestingly, IL-6 was also one of the most abundant cytokines present in endometrial explants, with a 3.7-fold increase in BB compared to U-IC samples, and its expression could be influenced by embryo presence. Supporting this, porcine embryos can modify cytokine signaling in their surrounding environment, and the expression of IL-6 increases in endometrial explants after their exposure to embryo-derived factors [37]. Similarly, it has been suggested that human blastocysts influence IL-6 signaling in their surroundings [38].

IL-10 is another anti-inflammatory pleiotropic cytokine that plays an important role in maternal-embryo immune tolerance mechanisms by reducing the inflammatory response at the uterine level, thus leading to the immunosuppressed state necessary for semi-allogenic conceptus acceptance [39]. Our results showed increased levels of this cytokine in explants of BB sows compared to those of U-IC sows (7.0-fold increase). Very recent reports pointed out the relevance of IL-10 in human pregnancy. Although increased production of this cytokine is associated with successful embryo implantation [40], low IL-10 expression has been related to a decrease in endometrial receptivity resulting in blastocyst implantation failures [41].

TGFβ family members are crucial signaling factors during embryo-maternal communication and pregnancy establishment in different animal species [42,43,44]. In pigs, the expression of TGFβ1, 2 and 3 has been identified in the pregnant endometrium and conceptus between days 10 and 14 of pregnancy [45]. Our results indicate that TGFβ production by the BB endometrium is already present at Day 6 of pregnancy, suggesting an earlier role of these cytokines. Interestingly, Day-6 porcine embryos modulate the TGFβ pathway through the regulation of SMAD2 gene expression [33]. This is an important aspect because TGFβs are directly related to the establishment of the anti-inflammatory response at the uterine level necessary to avoid embryo rejection [46].

IL-1α is a proinflammatory cytokine that belongs to the IL-1 family and has an important role in embryo-maternal communication during the implantation period in different species of mammals, including pigs [15]. Our results show that IL-1α levels were 3.6-fold higher in BB sows than in U-IC sows, which, as occurs with the other cytokines, might be due to the presence of embryos. The fact that IL-1α production is controlled by IL-1β [47] and IL-1β is secreted by Day-6 porcine embryos [15] suggests that the presence of early embryos could be the reason for the increased IL-1α levels in endometrial explants of BB sows. Interestingly, IL-1α is considered an alarm signal initiating the inflammatory response, which is up-regulated by several inflammatory cytokines, including IL-1β [48]. This inflammatory response could be countered by the increased levels of IL-1ra detected in BB sows, which would act as a protective agent of the endometrium and embryo against IL-1 proinflammatory action [35], as mentioned above.

IL-2, a pleiotropic cytokine with mainly proinflammatory functions [49], was more than 5-fold higher in BB than in U-IC sows. It could be speculated that such differences are related to the inflammatory response of the uterus to AI component stimuli, which are not present in U-IC sows, as a single exposure to semen triggers an inflammatory response of the uterus 4 days after mating involving, among other cytokines, the secretion of IL-2 [50].

Proinflammatory IL-8 was also more abundant in explants from BB sows (1.6-fold increase with respect to U-IC sows). Although no evidence about the modulation of this cytokine by porcine AI components and/or embryos is available, it has been described that IL-8 is up-regulated by the presence of normal human blastocysts, while in the absence of embryos, this effect does not appear [51].

The cytokine IFNγ was also increased in explants from BB sows (6.0-fold increase compared to U-IC sows). This cytokine is mainly produced by natural killer (NK) cells [52]. Interestingly, endometrial NK cells have elevated activity in pregnant gilts and very low activity in cyclic, pseudo-pregnant or animals inseminated with SP or dead spermatozoa [53], suggesting that embryo signaling is determinant for their activity. Therefore, the elevated levels of IFNγ found in samples from BB sows in the present study could be due to the high activity of NK cells in the BB endometrium described by these authors.

In the present study, 4 (2 anti- and 2 proinflammatory cytokines) out of the 16 cytokines analyzed were below the detection limit of the assay in explants from U-IC sows. Although these cytokines were not further statistically analyzed, these low levels are consistent with the tendency shown by the other cytokines, confirming the low cytokine production by the endometrial explants of U-IC sows.

From a practical point of view, it is important to know the characteristics of the uterine environment surrounding the embryos at this early preimplantation period to mimic these conditions in some reproductive technologies, such as ET. Under this premise, information about anti-/proinflammatory cytokine balance could precisely indicate the necessary conditions for optimal embryo survival and development. Our results indicate a predominant anti-inflammatory status at this time of the cycle, regardless of whether sows were pregnant or not. Furthermore, the shift to an anti-inflammatory status was more evident in BB sows (83.3% of the anti-inflammatory ratios evaluated were higher in BB than in U-IC sows). These results seem to indicate that at Day 6 of pregnancy, the porcine uterine environment has changed or is changing from a transient proinflammatory stage, derived from exposure to AI components, to a more permissive anti-inflammatory status, which might positively contribute to embryo–maternal immune tolerance [12,13,16].

## 5. Conclusions

In conclusion, our results indicate that the levels of most cytokines and the balance of anti- and proinflammatory cytokines were higher in endometrial explants of BB than in U-IC sows. Taking into account that cyclic sows are used as recipients in pig ET procedures and the importance of an adequate uterine immune environment for achieving optimal embryo development and pregnancy establishment, modifying the uterine cytokine profile of cyclic/recipient sows could be a useful tool for improving ET outcomes in pigs. Our findings suggest that AI components and/or the presence of embryos condition the uterine environment of BB sows by inducing an immune-tolerant cytokine profile at Day 6 of pregnancy. Further research is needed to determine whether the SP or some of its active components, in combination or separately, could be a useful tool for optimizing endometrial receptivity and embryo development, leading to a maximization of the efficiency of porcine ET technologies.

## Figures and Tables

**Table 1:** Ratios (mean ± SD) of anti-inflammatory to proinflammatory cytokines showing significant differences between endometrial explants of blastocyst-bearing (BB) and uninseminated cyclic (U-IC) sows.

Anti-/Proinflammatory Cytokine	Cytokine Ratio ^1^		Fold Increase ^2^	*p*-Value
	BB Sows	U-IC Sows		
IL-1ra/IL-1α	452.7 ± 299.6	182.7 ± 38.0	**2.5**	*p* < 0.001
IL-1ra/IL-1β	18.1 ± 14.2	4.0 ± 2.2	**4.5**	*p* < 0.001
IL-1ra/IL-8	2.6 ± 2.1	0.5 ± 0.2	4.8	*p* < 0.001
IL-1ra/IL-18	14.7 ± 12.6	3.2 ± 2.0	**4.6**	*p* < 0.001
IL-1ra/GM-CSF	312.6 ± 205.7	43.4 ± 22.4	**7.2**	*p* < 0.001
IL-6/IL-1β	14.7 ± 6.2	6.6 ± 3.1	**2.2**	*p* < 0.001
IL-6/IL-8	2.0 ± 0.7	0.9 ± 0.3	2.3	*p* < 0.001
IL-6/IL-18	11. 6 ± 4.4	6.0 ± 4.4	**1.9**	*p* < 0.001
IL-6/GM-CSF	276.6 ± 101.2	70. 6 ± 30.0	**3.9**	*p* < 0.001
IL-10/IL-1β	0.1 ± 0.05	0.05 ± 0.02	**1.9**	*p* < 0.01
IL-10/IL-2	0.4 ± 0.1	0. 6 ± 0.2	0.7	*p* < 0.01
IL-10/IL-8	0.01 ± 0.01	0.006 ± 0.003	1.9	*p* < 0.01
IL-10/IL-18	0.1 ± 0.02	0.03 ± 0.02	1.8	*p* < 0.001
IL-10/GM-CSF	1.5 ± 0.4	0.5 ± 0.2	3.3	*p* < 0.001
IL-10/IFNγ	0.01 ± 0.01	0.02 ± 0.01	0.6	*p* < 0.001
TGFβ1/IL-2	1.7 ± 0.6	3.3 ± 1.7	0.5	*p* < 0.001
TGFβ1/IL-8	0.05 ± 0.02	0.03 ± 0.01	1.5	*p* < 0.05
TGFβ1/IL-18	0.3 ± 0.2	0.2 ± 0.1	1.6	*p* < 0.05
TGFβ1/GM-CSF	6.3 ± 2.5	2.4 ± 0.7	**2.7**	*p* < 0.001
TGFβ1/IFNγ	0.04 ± 0.01	0.1 ± 0.1	0.4	*p* < 0.001
TGFβ2/IL-1β	1.0 ± 0.8	0.7 ± 0. 6	1.4	*p* < 0.05
TGFβ2/IL-2	4.5 ± 1.9	9.3 ± 5.8	0.5	*p* < 0.01
TGFβ2/IL-18	0.7 ± 0.3	0.4 ± 0.2	1.5	*p* < 0.05
TGFβ2/GM-CSF	16.2 ± 8.0	6.3 ± 2.5	**2.6**	*p* < 0.001
TGFβ2/IFNγ	0.1 ± 0.03	0.3 ± 0.2	0.4	*p* < 0.001

^1^ The anti-/proinflammatory ratio was calculated by dividing the levels of anti-inflammatory cytokines by the proinflammatory cytokines. Green and red indicate a shift towards an anti-inflammatory or proinflammatory profile, respectively. ^2^ Bold values indicate the fold increase of the anti-inflammatory ratio in BB sows compared to U-IC sows.
